# The effect of leucocytosis on retinopathy of prematurity

**DOI:** 10.1038/s41598-023-47298-z

**Published:** 2023-11-21

**Authors:** Zhihong Sun, Lu He, Congcong Zhao, Hongbo Zhang, Ping Cheng, Yingying Wang, Mingchao Li, Zengyuan Yu, Huiqing Sun

**Affiliations:** Department of Neonatology, Children’s Hospital Affiliated to Zhengzhou University, Henan Children’s Hospital, Zhengzhou Children’s Hospital, 33 Longhuwaihuan Road, Zhengzhou, 450018 Henan China

**Keywords:** Paediatric research, Retinal diseases

## Abstract

Postnatal leukocytosis reflects the general condition of inflammatory. Infection and inflammatory reaction have been proven to affect the occurrence of ROP and other visual dysfunction. Infants with a gestational age of < 28 weeks who were less than three days of age and admitted to the hospital between September 2015 and March 2021 were included in the study. Infants with a white blood cell (WBC) count ≥ 30 × 10^9^/L were assigned to the leucocytosis group (n = 82). Gestational age- and weight-matched infants without leucocytosis were included as a control group (n = 85). The incidence and prognosis of ROP in preterm infants were compared between the groups. Receiver operating characteristic (ROC) curves were used to analyse the correlation between the WBC count and severe ROP. Compared to the infants in the control group, those in the leucocytosis group had lower 1-min Apgar scores (*p* < 0.001); higher C-reactive protein (*p* < 0.001) and procalcitonin (*p* < 0.001); and higher incidences of intracranial haemorrhage (*p* = 0.007), leukomalacia (*p* = 0.045), sepsis (*p* = 0.006), bronchopulmonary dysplasia (*p* = 0.017). The maternal age was higher in the leucocytosis group (*p* < 0.001). After adjusting for gestational age at 45 weeks, the incidence of severe ROP (*p* = 0.001) and the requirement for ranibizumab injections (*p* = 0.004) were higher in the leucocytosis group. The cut-off WBC count was determined to be 19.1 × 10^9^/L, with a sensitivity of 88.6%, a specificity of 77.3%, and an area under the curve of 0.941 (95% confidence interval: 0.904–0.978) for the detection of severe ROP. Leucocytosis may be associated with severe ROP in premature infants.

## Introduction

Leucocytosis, a severely elevated white blood cell (WBC) count, occurs in approximately 1.3–17.0% of infants in the neonatal intensive care unit^1^ and can be caused by infection, inflammation, stress, or medications. Postnatal leucocytosis reflects an increased release of immature granulocytes in the bone marrow or foetal systemic inflammatory response syndrome. Chorioamnionitis may result in leucocytosis in premature infants. In addition, sepsis, hypoxia, asphyxia, and inflammatory reactions in premature infants after birth may also cause leucocytosis in premature infants. Leucocytosis significantly affects the prognosis of premature infants^2^.

Retinopathy of prematurity (ROP) is also common in premature infants. ROP is an abnormally proliferative disease of the retinal vasculature that can damage vision in premature infants and may result in blindness^3^. Recent studies have reported that infection and inflammatory reactions affect the occurrence of ROP and other diseases that affect vision^4^. However, an increase in the WBC count is not related to the occurrence and development of ROP^1, 5^, it may be an expression of the body's inflammatory response^6^. Such as may be associated with activation of the inflammatory cascade associated with preterm birth, RDS, cerebral white matter injury, IVH, NEC, BPD and cerebral palsy^7^. Inflammation plays an important role in increasing the risk of ROP^8^. Infections and inflammation are the most common causes of leucocytosis. Furthermore, Leucocytosis was associated with a lower gestational age (GA) (25.1 vs 25.6 weeks) and higher rates of maternal chorioamnionitis^9^. Thus, leucocytosis may indirectly reflect ROP; therefore, this study investigated the relationship between leucocytosis and ROP and evaluated if leucocytosis can be used to predict the occurrence of ROP.

## Materials and methods

### Patient population

Premature infants admitted to the neonatal intensive care unit between September 2015 and March 2021 who were born at a gestational age of < 28 weeks and were less than three days old were included in this retrospective cohort study. All patients underwent fundal screening at age 4–6 weeks or at a corrected gestational age of 32 weeks until a corrected gestational age of 45 weeks, as recommended in the Guidelines for Treatment of Oxygen and Prevention and Treatment of Retinopathy in Preterm Infants^10^. Infants with genetic metabolic diseases, congenital dysplasia, congenital heart disease, other retinopathies, or infections detected during blood testing were excluded from the study.

### Data collection

The infants’ sex, gestational age, birth weight, WBC count, and treatment and related complications including intracranial haemorrhage, leukomalacia, necrotizing enterocolitis, sepsis, bronchopulmonary dysplasia, and ROP were retrospectively retrieved from the medical records.

The infants were grouped according to their WBC count. A WBC count ≥ 30 × 10^9^/L was diagnosed as leucocytosis^1, 5^. Therefore, infants with a WBC count ≥ 30 × 10^9^/L were included in the leucocytosis group. Gestational age- and birth weight-matched premature infants without leucocytosis were included as a control group. Gestational age matched was defined as gestational age plus or minus 3 days, and birth weight matched was defined as plus or minus 50 g.

All patients underwent routine blood tests on day 0, day 3, including red blood cell, WBC, and platelet counts. The WBC count was divided into total WBCs, neutrophils, lymphocytes, eosinophils, basophils, and monocytes using an automatic routine blood test instrument xe5000 (Shanghai, China, Sysmex company). The WBC, granulocyte (neutrophils, lymphocytes, and basophils), and lymphocyte counts were collected within three days after birth^11^. According to the International Classification of ROP, the disease is categorized into five stages of severity. In stage 1 ROP, a white and flat dividing line is observed between the vascular area in the posterior pole of the retina and the surrounding non-vascular area. In stage 2 ROP, the white boundary line becomes wider and higher, forming a ridge higher than the surface of the retina. In stage 3 ROP, the cristae become more prominent and pink, accompanied by fibre proliferation. In stage 4 ROP, partial retinal detachment occurs. Stage 4A includes peripheral retinal detachment that does not affect the macula, while stage 4B includes peripheral retinal detachment that does involve the macula. In stage 5 ROP, the retina is completely detached and is often funnel-shaped. The funnel can be classified as wide or narrow. In this study, the presence of immature or mature vessels with normal formation was defined as no ROP. Severe ROP was defined as ROP requiring ophthalmic surgery, including any stage 1 ROP before the threshold, threshold ROP, and stage 3 or greater ROP^12^.

### Fundal screening

The recommendations included in the Guidelines for Treatment of Oxygen and Prevention and Treatment of Retinopathy in Preterm Infants (Revised Edition)^10^ were used during fundal screening and ROP treatment in this study. A Retcam III wide-angle digital retinal imaging system (Natus Medical Incorporated, California, USA) was used to conduct the fundal screening. All fundal screening examinations were conducted by a single, experienced ophthalmologist.

### Statistical methods

Continuous variables are expressed as mean ± SD or as median and range. Categorical variables are expressed as number and frequency. Continuous variables were compared using the t-test, while categorical variables were compared using the chi-squared or Fisher’s exact tests, as appropriate. For confounding factor analyses, logical regression analyses were used. The correlation between the WBC count and severe ROP was analysed using the receiver operator characteristic curve to predict the cut-off value of an abnormal WBC count. SPSS (version 21.0, IMB Corp., New York, USA) was used to conduct the analyses. Statistical significance was set at *P* < 0.05.

### Ethics approval and consent to participate

All methods in this study were carried out in accordance with the ethical standards as laid down in the 1964 Declaration of Helsinki and its later amendments or comparable ethical standards. The Life Science Ethics Committee of Children’s Hospital affiliated to Zhengzhou University approved the study. Written informed consent was obtained from all participants’ parents.

## Results

### Demographic data

During the study period, 430 premature infants born at a gestational age < 28 weeks were admitted to the neonatal intensive care unit, including 105 with a WBC count ≥ 30 × 10^9^/L. Nine patients with incomplete data, six for whom treatment was discontinued, five who died, and three with congenital malformations or genetic metabolic diseases were excluded from the study. The final analysis included 82 premature infants with leucocytosis (50 males and 32 females) born at a mean gestational age of 26.9 ± 1.8 weeks (range: 24–28 weeks). The mean birth weight was 1110 ± 212 g (range: 540–1500 g). The control group included 85 patients (49 males and 46 females), with a mean gestational age of 27.1 ± 1.7 weeks (range: 24–28 weeks). The mean birth weight was 1107 ± 221 g (range: 550–1490 g). The baseline clinical characteristics of the two groups were not significantly different (Table 1).

**Table 1:** Comparison of baseline data of the two groups.

	Control group (N = 85)	Leukocytosis group (N = 82)	Statistical values	*P* value
Birth weight b	1107 ± 221	1110 ± 212	0.092	0.929
Gestational age b	27.1 ± 1.7	26.9 ± 1.8	0.741	0.461
Male a	49 (57.6)	50 (61.0)	0.192	0.753
Cesarean section a	51 (60.0)	56 (68.3)	1.247	0.333
Age at enrollment b	48.7 ± 14.3	45.8 ± 13.2	1.361	0.176

a is represented by example (%), the statistical value is χ2 value, and b is represented by the statistical value is the t value.

### Clinical data

Compared to infants in the control group, those in the leucocytosis group had lower 1 min Apgar scores (*p* < 0.001); higher C-reactive protein (*p* < 0.001) and procalcitonin (*p* < 0.001); and higher incidences of intracranial haemorrhage (*p* = 0.007), leukomalacia (*p* = 0.045), sepsis (*p* = 0.006), and bronchopulmonary dysplasia (*p* = 0.017). The maternal age was higher in the leucocytosis group (*p* < 0.001) (Table 2).

**Table 2:** Comparison of clinical data of the two groups.

	Control group N = 85	Leukocytosis group N = 82	Statistical values	*P* value
Maternal age (y) b	27.8 ± 5.8	35.5 ± 6.3	8.223	< 0.001
Conception by ART a	5 (5.9)	14 (17.1)	5.184	0.028
Chorioamnionitis a	7 (8.2)	18 (22.0)	6.168	0.017
Apgar score (1 min) b	7.3 ± 2.2	5.3 ± 2.1	6.012	< 0.001
RDS a	65 (76.5)	72 (87.8)	3.638	0.070
BPD a	5 (5.9	15 (18.3)	6.098	0.017
NEC a	3 (3.5)	7 (8.5)	1.700	0.329
IVH (≥ stage III) a	4 (4.7)	15 (18.3)	7.641	0.007
Leukomalacia a	3 (3.5)	10 (12.2)	4.366	0.045
Sepsis a	16 (18.8)	33 (40.2)	8.411	0.006
Severe ROP a	10 (11.8)	28 (34.1)	11.900	0.001
CRP b	8.1 ± 9.2	15.1 ± 10.5	4.594	< 0.001
PCT b	0.4 ± 0.2	3.2 ± 3.4	7.582	< 0.001

RDS is respiratory distress syndrome, BPD is bronchopulmonary dysplasia, IVH is intracranial hemorrhage, NEC is necrotizing enterocolitis, CRP is C-reactive protein, PCT is procalcitonin; a is represented by example (%), the statistical value is χ2 value, and b is represented by the statistical value is the t value.

### Risk factor analysis

Leucocytosis was identified as a risk factor for severe ROP (statistical information; Table 3).

**Table 3:** Logistic regression analysis of confounding factors for the development of retinopathy of severe prematurity.

	B	S.E	Forest	Itself	Exp(B)	95% CI for EXP(B)
Maternal age	0.008	0.131	0.003	0.953	1.008	0.780–1.302
Conception by ART	0.268	2.446	0.012	0.913	1.307	0.011–157.890
Chorioamnionitis	1.113	2.362	0.222	0.637	3.044	0.030–312.159
Leukocytosis	1.285	0.608	4.474	0.034	3.616	1.099–11.897
RDS	− 14.626	15.560	0.000	0.991	0.065	0.021–1.224
BPD	0.470	1.033	0.207	0.649	1.600	0.211–12.105
IVH	− 0.291	0.844	0.119	0.730	0.748	0.143–3.913
PVL	19.468	21.156	0.000	0.993	2.849	0.035–1.311
septicemia	− 0.163	2.266	0.005	0.943	0.849	0.010–72.152
Apgar score(1 min)	− 0.092	0.108	0.727	0.394	0.912	0.737–1.128
CRP	0.140	0.110	1.621	0.203	1.150	0.927–1.426
PCT	1.871	2.042	0.840	0.359	6.497	0.119–355.470

*CI* confidence interval; *SE* Standard error; *Wald* wald test; *RDS* respiratory distress syndrome; *BPD* bronchopulmonary dysplasia; *IVH* intracranial hemorrhage; *PVL* leukomalacia; *CRP* C-reactive protein; *PCT* procalcitonin.

### ROP

**Figure 1:**
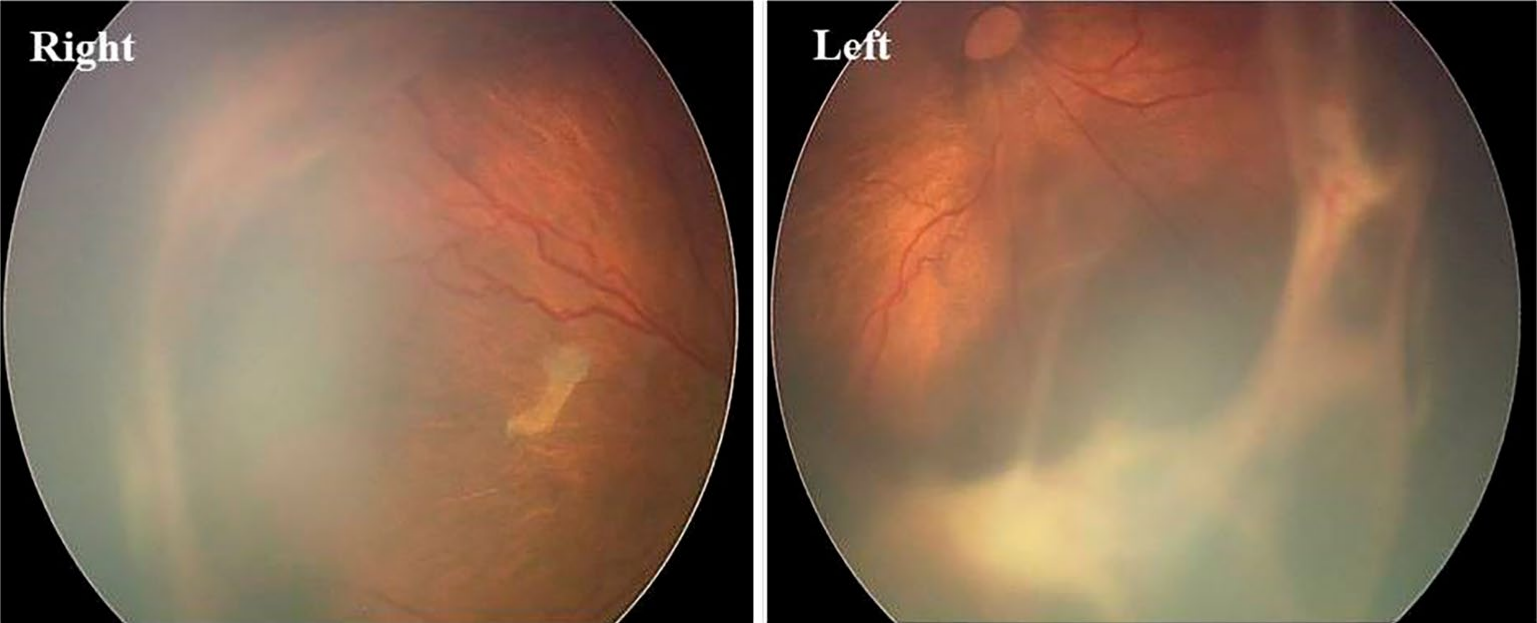
A girl, gestational age 30 weeks, birth weight 1200 g, severe ROP at 42 weeks of corrected gestational age, stage 4 AOP (partial retinal detachment) in the right eye and stage 5 ROP (total retinal detachment) in the left eye.

At a corrected gestational age of 45 weeks, the incidence of severe ROP was significantly higher in the leucocytosis group than in the control group (*p* = 0.001) (Table 4 and Fig. 1). The use of laser therapy was not significantly different between the groups. The number of premature infants requiring ranibizumab monoclonal antibody injections was significantly higher in the leucocytosis group than in the control group (*p* < 0.05).

**Table 4:** Comparison of severe ROP between leukocytosis group and control group at 45 weeks of gestational age correction.

	Control group (n = 85)	Leukocytosis group (n = 82)	× 2	*P* value
Severe ROP				
Patient	10 (11.8)	28 (34.1)	11.9	0.001
Eyes (only)	18 (10.6)	51 (31.1)	21.421	< 0.001
Laser treatment				
Patient	4 (4.7)	9 (11.0)	2.286	0.156
Eyes (only)	7 (4.1)	15 (9.1)	3.431	0.078
Ranibizumab injection therapy				
Patient	6 (7.1)	19 (23.2)	8.512	0.004
Eyes (only)	11 (6.5)	36 (22.0)	19.784	< 0.001

### Correlation between leucocytosis and severe ROP

**Figure 2:**
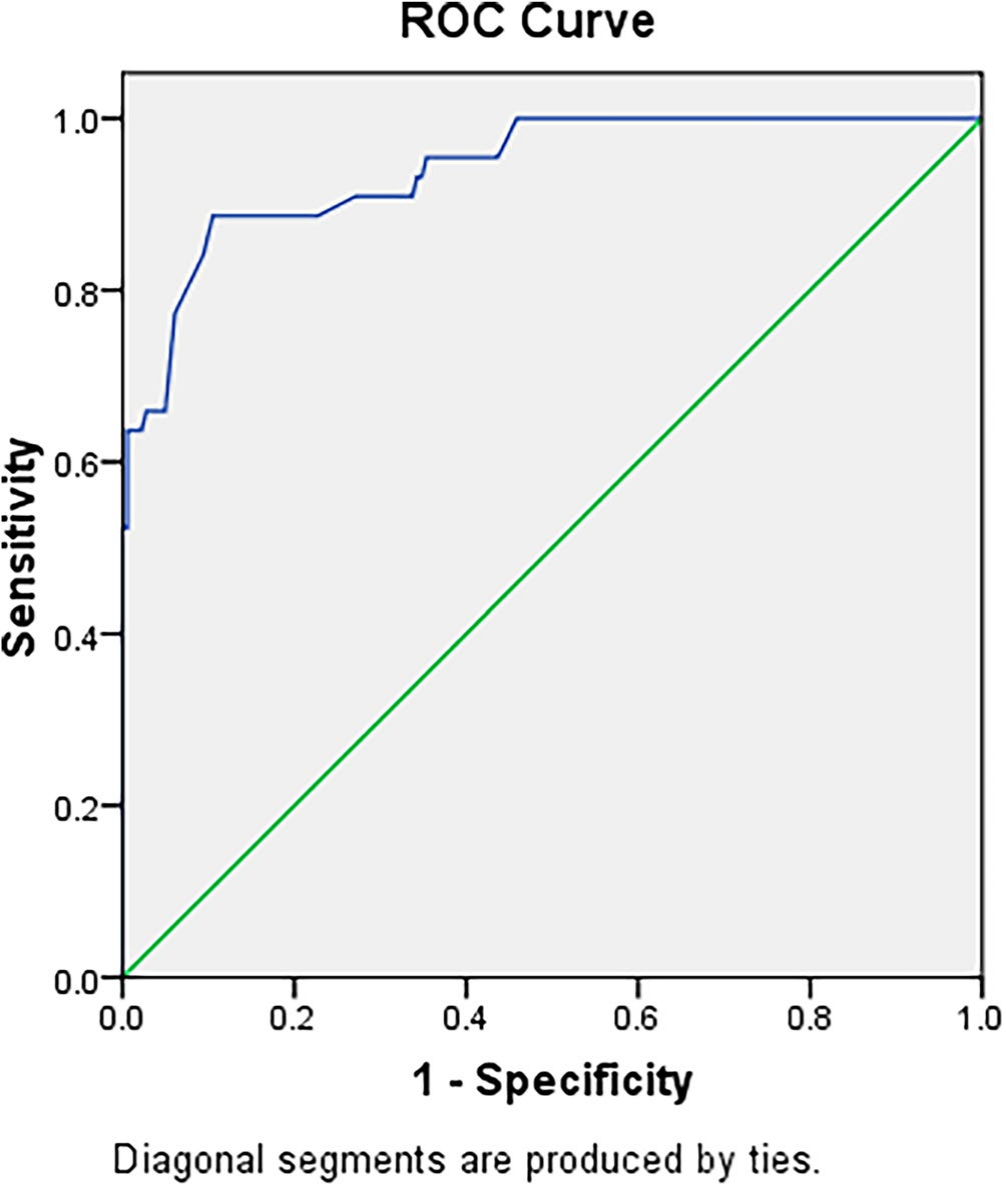
Working characteristic curve of white blood cell count and subject with severe retinopathy of prematurity.

The cut-off value of an abnormal WBC count was 19.1 × 10^9^/ L, with a sensitivity of 88.6%, a specificity of 77.3%, and an area under the curve of 0.941 (95% confidence interval 0.904–0.978) (Fig. 2).

## Discussion

This study aimed to raise awareness among neonatologists about potentially considering leucocytosis a means for early identification of severe ROP. We selected WBC in this study as a predictor owing to its wide use in clinical settings; this will further promote effortless application of this screening process among neonatologists.

Many factors affect WBC count including infection, inflammation, stress, and medications^2^. The incidence of confirmed infection among infants with leucocytosis is 22%^1^, which is similar to that in adults^13^. Healthy new-borns and premature infants have a peak WBC count after birth^14^ which may be due to an increase in the number of cells in the marginal granulocyte pool mediated by catecholamines or the production and release of immature granulocytes in the bone marrow induced by postpartum stress.

There are a number of factors that can lead to elevated leukocytes, infection being one of the most common. Clinical chorioamnionitis leads to elevated white blood cells and fetal inflammatory response syndrome (FIRS), and it has been shown that babies born to mothers with histologic and clinical chorioamnionitis have a higher incidence of ROP^15, 16^. Prenatal administration of steroids reduces the risk of ROP development and progression to severe ROP, but at the same time higher leukocyte counts were observed in newborns from postnatal day 7 to day 14, probably achieved by reducing the excessive inflammatory response, which can still be realized by the production of inflammatory factors, inducing endothelial growth factor, when excess leukocytes are present in leukocytosis^17, 18^. In addition, transient myelopoietic abnormalities due to Down's syndrome are also a factor in leukocytosis^19^. Inflammatory mediators stimulate monocytes and macrophages to produce granulocyte colony-stimulating factor, increasing the WBC count^20^. Further research is required to determine whether the association between leucocytosis and ROP can be attributed to infection, inflammation, or stress.

Prenatal and postnatal inflammation may be key factors in the initiation and development of ROP, which may contribute to ROP either individually or concurrently^21^. While maternal infections lead to reduced immunity in the newborn and increase the possibility of infection and inflammatory storms^22^, sepsis in the infant leads to decreased IGF-1 (insulin-like growth factor-1)^23^. A previous study suggested that IGF-1 deficiency is related to insufficient vascular growth and subsequent proliferative ROP^24^.

During the first stage of ROP, inflammation inhibits the development of retinal nerves and vessels, leading to increased vascular occlusion resulting in increased retinal hypoxia. These factors promote abnormal neovascularization during the second stage of ROP and increase the risk of severe ROP^4^. Inflammation may increase the risk of ROP by sensitizing the developing retina and neovascularization to oxygen-induced growth factors. In contrast, the stress state of the retina can promote the activation of inflammatory cells and the production of inflammatory cytokines, ultimately affecting neovascularization^4^.

Increased levels of inflammatory biomarkers, including cytokines and C-reactive protein, during the first few weeks after birth, are associated with the development of ROP at a later stage^25^. A previous study reported that histologic chorioamnionitis and funisitis are unrelated to the occurrence and development of ROP, though maternal chorioamnionitis and leucocytosis may increase the risk of ROP^26^. In this study, the incidence of severe ROP in infants with leucocytosis was significantly higher than that in the control group, and the cut-off WBC count had high sensitivity, specificity, and area under the curve.

Ashki et al.^27^ reported that the infiltration of macrophages, monocytes, and leukocytes led to the release of nitric oxide (NO) and superoxide anions from tissues, resulting in the conversion of NO to peroxynitrite, leading to an increase in angiogenic growth factors, such as vascular endothelial growth factor, basic fibroblast growth factor, and hypoxia-inducible factor. This mechanism may also be involved in the development of ROP^28^.

The lymphocyte-to-monocyte ratio (LMR) and monocyte counts in preterm infants at 4 weeks postnatally are independent risk factors for the development of ROP. Increased WBC and neutrophil counts increase the risk of ROP and the LMR can be used to predict the occurrence of ROP within 24 h after birth^29, 30^. The NLR was not a risk factor for ROP development but was a risk factor for ROP treatment^31^. In this study, a WBC ≥ 30 × 10^9^/L was closely related to the increased risk of severe ROP in ultra-preterm infants and may be a predictor of severe ROP in this population.

## Conclusions

The WBC count is a simple, economical, and widely used clinical parameter. Neonatologists can use the WBC count to predict the development of severe ROP, allowing for timely screening evaluations and interventions, which will ultimately reduce the risk of developing severe ROP. A prospective study with a larger patient population is required to further investigate the correlation between leucocytosis and severe ROP.

## Data Availability

The datasets used and/or analyzed during the current study are included in this published article.

## References

[1] Morag I, Dunn M, Nayot D, Shah PS (2008). Leukocytosis in very low birth weight neonates: Associated clinical factors and neonatal outcomes. J. Perinatol..

[2] Juul SE, Haynes JW, McPherson RJ (2004). Evaluation of neutropenia and neutrophilia in hospitalized preterm infants. J. Perinatol..

[3] Higgins RD (2019). Oxygen saturation and retinopathy of prematurity. Clin. Perinatol..

[4] Lee J, Dammann O (2012). Perinatal infection, inflammation, and retinopathy of prematurity. Semin. Fetal Neonatal Med..

[5] Duran R, Ozbek UV, Ciftdemir NA, Acunaş B, Süt N (2010). The relationship between leukemoid reaction and perinatal morbidity, mortality, and chorioamnionitis in low birth weight infants. Int. J. Infect. Dis..

[6] Borțea CI, Enatescu I, Dima M, Pantea M, Iacob ER, Dumitru C (2023). A prospective analysis of the retinopathy of prematurity correlated with the inflammatory status of the extremely premature and very premature neonates. Diagnostics (Basel).

[7] Hsiao R, Omar SA (2005). Outcome of extremely low birth weight infants with leukemoid reaction. Pediatrics.

[8] Kong L, Demny AB, Sajjad A, Bhatt AR, Devaraj S (2016). Assessment of plasma cytokine profile changes in bevacizumab-treated retinopathy of prematurity infants. Invest. Ophthalmol. Vis. Sci..

[9] Lundgren P, Klevebro S, Brodin P, Smith LEH, Hallberg B, Hellström A (2019). Leucocytosis is associated with retinopathy of prematurity in extremely preterm infants. Acta Paediatr..

[10] Li Q, Zhang G, Feng Z (2016). Interpretation of guidelines for treatment of oxygen and prevention and treatment of retinopathy in preterm infants. J. Dev. Med..

[11] International Committee for the Classification of Retinopathy of P (2005). The international classification of retinopathy of prematurity revisited. Arch. Ophthalmol..

[12] Sun H, Kang W, Cheng X, Chen C, Xiong H, Guo J (2013). The use of the WINROP screening algorithm for the prediction of retinopathy of prematurity in a Chinese population. Neonatology.

[13] Portich JP, Faulhaber GAM (2020). Leukemoid reaction: A 21st-century cohort study. Int. J. Lab. Hematol..

[14] Xanthou M (1970). Leucocyte blood picture in healthy full-term and premature babies during neonatal period. Arch. Dis. Child..

[15] Ajayi SO, Morris J, Aleem S, Pease ME, Wang A, Mowes A (2022). Association of clinical signs of chorioamnionitis with histological chorioamnionitis and neonatal outcomes. J. Matern. Fetal Neonatal Med..

[16] Galinsky R, Polglase GR, Hooper SB, Black MJ, Moss TJ (2013). The consequences of chorioamnionitis: Preterm birth and effects on development. J. Pregnancy.

[17] Yim CL, Tam M, Chan HL, Tang SM, Au SCL, Yip WWK (2018). Association of antenatal steroid and risk of retinopathy of prematurity: A systematic review and meta-analysis. Br. J. Ophthalmol..

[18] Peng CT, Lin HC, Lin YJ, Tsai CH, Yeh TF (1999). Early dexamethasone therapy and blood cell count in preterm infants. Pediatrics.

[19] Kuo E, Kumarapeli AR (2020). Placental pathology in down syndrome-associated transient abnormal myelopoiesis. Arch. Pathol. Lab. Med..

[20] Calhoun DA, Kirk JF, Christensen RD (1996). Incidence, significance, and kinetic mechanism responsible for leukemoid reactions in patients in the neonatal intensive care unit: A prospective evaluation. J. Pediatr..

[21] Dammann ORJ, Chemtob S (2021). The prenatal phase of retinopathy of prematurity. Acta Paediatr..

[22] Woo SJ, Park KH, Lee SY, Ahn SJ, Ahn J, Park KH (2013). The relationship between cord blood cytokine levels and perinatal factors and retinopathy of prematurity: A gestational age-matched case-control study. Invest. Ophthalmol. Vis. Sci..

[23] Ashare A, Nymon AB, Doerschug KC, Morrison JM, Monick MM, Hunninghake GW (2008). Insulin-like growth factor-1 improves survival in sepsis via enhanced hepatic bacterial clearance. Am. J. Respir. Crit. Care Med..

[24] Chen J, Smith LEH (2007). Retinopathy of prematurity. Angiogenesis.

[25] Holm M, Morken TS, Fichorova RN, VanderVeen DK, Allred EN, Dammann O (2017). Systemic inflammation-associated proteins and retinopathy of prematurity in infants born before the 28th week of gestation. Invest. Ophthalmol. Vis. Sci..

[26] Woo SJ, Park KH, Jung HJ, Kim S, Choe G, Ahn J (2012). Effects of maternal and placental inflammation on retinopathy of prematurity. Graefes Arch. Clin. Exp. Ophthalmol..

[27] Ashki N, Chan AM, Qin Y, Wang W, Kiyohara M, Lin L (2014). Peroxynitrite upregulates angiogenic factors VEGF-A, BFGF, and HIF-1alpha in human corneal limbal epithelial cells. Invest. Ophthalmol. Vis. Sci..

[28] Vinekar A, Nair AP, Sinha S, Vaidya T, Chakrabarty K, Shetty R (2021). Tear fluid angiogenic factors: Potential noninvasive biomarkers for retinopathy of prematurity screening in preterm infants. Invest. Ophthalmol. Vis. Sci..

[29] Hu YX, Xu XX, Shao Y, Yuan GL, Mei F, Zhou Q (2017). The prognostic value of lymphocyte-to-monocyte ratio in retinopathy of prematurity. Int. J. Ophthalmol..

[30] Obata S, Matsumoto R, Kakinoki M, Sawada O, Sawada T, Saishin Y (2023). Association between treatment for retinopathy of prematurity and blood monocyte counts. Jpn. J. Ophthalmol..

[31] Obata S, Matsumoto R, Kakinoki M, Sawada O, Sawada T, Saishin Y (2023). Blood neutrophil-to-lymphocyte ratio as a risk factor in treatment for retinopathy of prematurity. Graefes Arch. Clin. Exp. Ophthalmol..

